# Breathless and awaiting diagnosis in UK lockdown for COVID-19…We’re stuck

**DOI:** 10.1038/s41533-021-00232-0

**Published:** 2021-05-05

**Authors:** Gillian Doe, Stacey Chantrell, Marie Williams, Michael C. Steiner, Natalie Armstrong, Ann Hutchinson, Rachael A. Evans

**Affiliations:** 1 Department of Respiratory Science, University of Leicester, Leicester, UK; 2 NIHR Biomedical Research Centre–Respiratory theme, University Hospitals of Leicester NHS Trust, Leicester, UK; 3 Allied Health and Human Performance, University of South Australia, Adelaide, Australia; 4 Department of Health Sciences, University of Leicester, Leicester, UK; 5 Wolfson Palliative Care Research Centre, Hull and York Medical School, University of Hull, Hull, UK

**Keywords:** Health services, Medical research, Diagnosis

## Abstract

During the COVID-19 pandemic, semi-structured interviews were undertaken with 20 adults awaiting a diagnosis for their chronic breathlessness. Three key themes were identified using thematic analysis: (1) de-prioritisation of diagnosis, (2) following UK ‘lockdown’ guidance for the general population but patients fearful they were more at risk, and (3) the impact of lockdown on coping strategies for managing breathlessness. The existing unpredictable pathway to diagnosis for those with chronic breathlessness has been further interrupted during the COVID-19 pandemic.

In March 2020, the World Health Organisation (WHO) declared a global pandemic of coronavirus disease (COVID-19) caused by severe acute respiratory syndrome coronavirus-2 (SARS-CoV-2). At the time of study conduct, there was no available vaccine so public health policy was reliant on reducing the transmission^1^. The UK entered national lockdown on 23rd March 2020 with specific government guidance including the closure of non-essential services and social distancing measures. Additional advice with support to ‘shield’ i.e., to stay at home and minimise face-to-face contact with others, was provided for those at risk of worse outcomes identified by coded diagnoses from healthcare records^1^. High-risk groups included people with severe respiratory or heart disease^1,2^.

People living with chronic breathlessness who were yet to receive a diagnosis for their underlying condition were not identified as clinically vulnerable so did not receive shielding advice or support; however, breathlessness is a common manifestation of severe heart and lung diseases^3^ so many may have been classified as ‘extremely vulnerable’ if they had received a diagnosis. Furthermore, functional impairment from breathlessness (assessed by the Medical Research Council dyspnoea scale) is associated with reduced survival regardless of underlying diagnosis and therefore may independently be an indicator for worse outcomes with COVID-19^4,5^.

Breathlessness is common and surveys of the general population indicate the prevalence is around 9–11%^6,7^, but people often delay seeking help until everyday activities become significantly impaired. Adults presenting with chronic breathlessness frequently experience significant delays in diagnosis and therefore treatment^8,9^. The reasons are multi-factorial and include the complex multimorbidity of breathlessness^10,11^, accessibility of investigations^12^, and variable adherence to disease-specific diagnostic pathways^9^.

We aimed to understand the experiences and impact of lockdown for adults who had presented with breathlessness to primary care but were yet to receive a diagnosis.

Twenty participants were interviewed between April–May 2020: 12 female, mean (range) age of 65 (45–89) years. Sixteen participants lived in a rural setting and four in the city, five participants lived alone. All participants were retired except for two participants who remained working throughout the lockdown period. The mean (range) number of comorbidities for the patients was 4 (0–10). None of those interviewed had been advised to shield. As highlighted in Fig. 1 two participants were interviewed shortly after the lockdown was eased and were included as their diagnostic process was potentially impacted. No distinct new codes were identified.

**Fig. 1:**
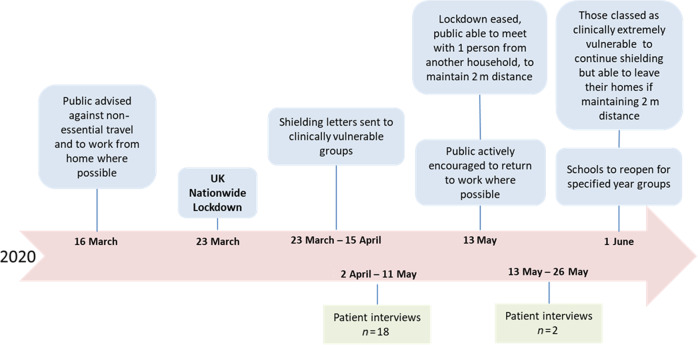
Interviews timeline. Timeline of the interviews in relation to government guidance and the lockdown period.

Three key themes were identified: (1) de-prioritisation of diagnosis by patients and healthcare system, (2) following UK ‘lockdown’ guidance for the general population but patients fearful they were more at risk, and (3) the impact of lockdown on coping strategies for managing breathlessness. Table 1 contains illustrative quotes.

**Table 1:** Results: Themes, description and quotes.

Theme	Quotes
1. De-prioritisation of diagnosis by patients and healthcare.	“I ain’t getting any healthcare at the moment. You know, I won’t bother the doctors with this at the moment because they won’t, I suppose they can’t do a lot over the telephone. And I wouldn’t want to go doctors to be seen because I wouldn’t, there’s a risk of catching anything.”
Description: The COVID-19 pandemic has led to a reduction in this group seeking healthcare; either appointments or investigations cancelled or no onward referral. Some described their breathlessness as a non- urgent problem and others felt worried about burdening their GP and the NHS at this time.	“Well, I was trying to just wait my turn sort of thing, I don’t really, they’re busy enough as it is aren’t they? If I cannot add to it, I won’t.”
	“I mean really to get a phone call from the doctor now is like asking for a miracle really anyway, and then you’re just going to say well I need some advice on my breathing. It feels wrong to even do that you see.”
	“…it’s inhibited me from going to see my GP to try and get kind of follow-up on what’s going on.”
	“Yeah they just said they’ll contact me when it’s possible to start doing things again, you know, because it’s not an urgent thing.”
	“So they phoned me… so she said… we’ll just leave it until the lockdown is finished.”
2. Following UK ‘lockdown’ guidance for the general population but fearful they are more at risk	“It feels like you’re being a bit of a bother for nothing, because I’ve not been actually diagnosed you see. We, who haven’t been diagnosed, are sort of out of the picture if you know what I mean, because we don’t get the letter for being vulnerable either… So we’re stuck.”
Description: This group were not identified as vulnerable and were following guidance for the general population. However, many describe a clear perception of being at increased risk if they were to contract COVID-19. Not being included in an ‘at risk’ group caused increased anxiety and uncertainty.	“So and I suppose in a separate way it’s more of a concern to me that if I am asthmatic, you know, if I was to get the COVID-19, would I be more at risk.”
	“General guidance, yes, that helps, but mostly for my health. I’m maybe a little bit frightened in case, I think if I got it I wouldn’t get over it because of my breathing. And yes when you can’t get your breath it is frightening, so I think that’s, obviously I don’t want to go just yet so.”
	“Because I have said to my husband, if I get this, it’s going to be serious because I have problems breathing anyway.”
3. Impact of lockdown on coping strategies for managing breathlessness.	Engaged Coping:
	“Yeah I’ve got a lady who comes on the tablet and she does yoga with me, tells me what to do. I do that twice a week.”
Description: People have expressed modified behaviour to learn new skills or change their routine to help them cope with lockdown. Some expressed not coping in the new environment. The nature of lockdown and reduced activity and social interaction reinforces the negative cycle of physical inactivity often seen with breathlessness.	“But I also make time now for a short walk, either in the morning or in the afternoon, just around the block.”
	“I mean like I said I’m doing exercises nearly every day and practising yoga and it’s just because I’m at home so I have time to do that. I don’t know what’s going to happen when I start work properly.”
	Disengaged coping:
	“It is very depressing not being able to go out anywhere. Even though I can’t walk
	that far without getting out of breath, I could still visit people, you know… it’s just being indoors all the time.”
	“Well since it’s been like it is, I’ve not been getting up very early and sometimes it’s, most days I don’t really want to get up. But, and sometimes I have to force myself.”
	“Well, I’m stuck in with this isolation thing which is driving me mad... and I find the less you do, the less you can do. So I can’t even go in the garden or do the gardening. But, you know, I potter round the house, I feed myself, I do the washing and that.”

People with chronic breathlessness awaiting a diagnosis described their experiences of the first UK lockdown due to the COVID-19 pandemic. We report de-prioritisation of seeking a diagnosis by both patients and healthcare systems. People perceived they were at greater risk than the general population yet were not receiving specific shielding guidance or support. A range of coping strategies were highlighted but people identified that attempts to keep active and contact with others were severely limited by the lockdown situation.

Long delays to diagnosis and therefore treatment is well documented in conditions associated with chronic breathlessness such as chronic obstructive pulmonary disease (COPD)^8^, heart failure^9^ and pulmonary fibrosis^13^. Unfortunately, our data highlights further delays to a diagnosis for those with breathlessness during the COVID-19 pandemic due to both patient behaviours and the healthcare system. Our data indicates many patients perceived their problems as less important when balanced with the effects of the COVID-19 pandemic, and understood why there were interruptions in their care. There was some anxiety around the risk of exposure by visiting a GP surgery. However, it was unclear when or how patients would be able to resume seeking help for their breathlessness.

Investigations for people with breathlessness commonly include spirometry, imaging and blood tests^14^. Many of these procedures were paused during the COVID-19 pandemic and the availability of spirometry was extremely limited due to the classification as an aerosol-generating procedure in UK national guidance^15^. Some patients also reported an initial delay in having blood tests but these were available once practices had appropriate measures and personal protective equipment (PPE) in place. The further delays to diagnosis may have harmful consequences such as causing patients to delay seeking help again until their symptoms are more disabling or at crisis point, halting the process altogether for some patients, and potentially delaying the access to effective therapy. It is currently unclear how the healthcare system will resume these services and deal with pre-existing requests.

Patients were concerned they were at higher risk than the general population from COVID-19 and it is likely that many people with chronic breathlessness would be categorised in a higher risk group for COVID-19 once a diagnosis is confirmed^16^. The quotes included in Theme 2 (Table 1) indicate an element of fear and anxiety from patients of becoming very unwell or dying if they contracted COVID-19. For future waves of COVID-19 or local outbreaks, people with severe functional limitation due to breathlessness may need to be considered as high risk with shielding guidance to incorporate people who have yet to receive a diagnosis or seek help.

Participants in this study described how the COVID-19 pandemic and the lockdown situation had altered their usual coping mechanisms. The concept of ‘Breathing Space’ is a combination of how people cope and seek help for their breathlessness, and how healthcare professionals respond to the needs of the person^17^. This concept encompasses engaged and disengaged coping styles^18^ including aspects such as problem-solving, social support, problem avoidance and social isolation, exemplified by participants in this study. Notably, the lockdown situation appeared to limit ‘Breathing space’ for many, as the usual ways of managing their mental and physical health such as going out, socialising and exercise were now severely curtailed, as were their opportunities to seek help for their breathlessness. The interruption in the diagnostic pathway highlighted a lack of symptom management from initial consultations. Irrespective of the underlying disease and availability of a diagnosis, there are evidence-based, effective, low-cost/risk non-pharmacological strategies available to help manage breathlessness^11^.

Our data shows people living with chronic breathlessness but without an established diagnosis are concerned they are at higher risk from COVID-19 and were unable to receive the same level of support as those ‘shielding’. Their healthcare has been interrupted by the COVID-19 pandemic causing further delays in an already unpredictable and long pathway to diagnosis, and methods they employ to cope with their breathlessness symptoms were compromised by the lockdown situation.

Patients and clinicians need to proactively re-engage with the pathway to diagnosis, treatment and management of chronic breathlessness. Despite challenges to ensure access to healthcare including diagnostic services, there remain opportunities to support patients to manage their symptoms regardless of the diagnosis.

## Methods

Semi-structured interviews were conducted with participants enrolled within a mixed-method feasibility study in Leicestershire, England: Breathlessness–DiagnosE Early in Primary care (Breathe-DEEP) with eligibility criteria of adults over forty years old, breathlessness for longer than two months, presenting for the first time and with no prior diagnoses accounting for their symptoms. The feasibility trial recruitment started in November 2019; patients within six months of presenting to their GP with breathlessness and willing to participate in an interview were eligible for this study. All participants provided written informed consent. The original interview guide was expanded to incorporate the pandemic situation; existing topic areas included experiences of breathlessness, related healthcare, and the larger research study. For this report, only data relevant to the impact of the COVID-19 lockdown experience on the diagnostic process were included. Figure 1 shows a timeline of the lockdown period and when these interviews were completed.

Interviews were conducted via telephone by one of two interviewers, who were trained in qualitative research methods, and transcribed verbatim. The transcripts were evaluated using thematic analysis^19^ supported by NVivo software. The analysis process included familiarisation with data, generating initial codes, searching for themes, reviewing themes, defining and naming themes and producing the report. Initial coding was carried out independently by two researchers and all interviews were reviewed by another member of the team. The research team discussed and reviewed the emerging themes throughout the data analysis using quotes from the transcripts to check data interpretation. For the third emerging theme relating to coping, Tobin’s categorisation of coping^18^ was used along with the concept of ‘Breathing Space’^17^ to analyse the patient’s descriptors of their coping mechanisms.

Research Ethics Committee Nottingham 1 provided ethical approval for the mixed methods Breathe DEEP trial which is the wider basis of this qualitative work. REC Reference: 19/EM/0201.

### Reporting summary

Further information on research design is available in the Nature Research Reporting Summary linked to this article.

## Supplementary information

Reporting Summary

## Data Availability

The data sets generated and analysed during this study are available from the corresponding author on reasonable request.
