# Supplementary material for: Breathless and awaiting diagnosis in UK lockdown for COVID-19…We’re stuck
Source: NPJ Prim Care Respir Med. 2021 May 5;31:21. doi: 10.1038/s41533-021-00232-0 (PMC8100135; doi:10.1038/s41533-021-00232-0)
Supplement: Supplementary file 1 — Reporting Summary [file 41533_2021_232_MOESM1_ESM.pdf]

# nature research

Corresponding author(s): Dr Rachael A Evans



# Reporting Summary

Nature Research wishes to improve the reproducibility of the work that we publish. This form provides structure for consistency and transparency in reporting. For further information on Nature Research policies, see Authors & Referees and the Editorial Policy Checklist.

## Statistical parameters

When statistical analyses are reported, confirm that the following items are present in the relevant location (e.g. figure legend, table legend, main text, or Methods section).

| n/a | Confirmed | |
|---|---|---|
| ☐ | ☒ | The exact sample size (*n*) for each experimental group/condition, given as a discrete number and unit of measurement |
| ☒ | ☐ | An indication of whether measurements were taken from distinct samples or whether the same sample was measured repeatedly |
| ☒ | ☐ | The statistical test(s) used AND whether they are one- or two-sided<br>*Only common tests should be described solely by name; describe more complex techniques in the Methods section.* |
| ☒ | ☐ | A description of all covariates tested |
| ☒ | ☐ | A description of any assumptions or corrections, such as tests of normality and adjustment for multiple comparisons |
| ☒ | ☐ | A full description of the statistics including central tendency (e.g. means) or other basic estimates (e.g. regression coefficient) AND variation (e.g. standard deviation) or associated estimates of uncertainty (e.g. confidence intervals) |
| ☒ | ☐ | For null hypothesis testing, the test statistic (e.g. *F*, *t*, *r*) with confidence intervals, effect sizes, degrees of freedom and *P* value noted<br>*Give P values as exact values whenever suitable.* |
| ☒ | ☐ | For Bayesian analysis, information on the choice of priors and Markov chain Monte Carlo settings |
| ☒ | ☐ | For hierarchical and complex designs, identification of the appropriate level for tests and full reporting of outcomes |
| ☒ | ☐ | Estimates of effect sizes (e.g. Cohen's *d*, Pearson's *r*), indicating how they were calculated |
| ☒ | ☐ | Clearly defined error bars<br>*State explicitly what error bars represent (e.g. SD, SE, CI)* |

*Our web collection on statistics for biologists may be useful.*

## Software and code

Policy information about availability of computer code

| | |
|---|---|
| Data collection | No software was used. |
| Data analysis | NVivo 12 was used to organise the interview data as part of the thematic analysis process. |

For manuscripts utilizing custom algorithms or software that are central to the research but not yet described in published literature, software must be made available to editors/reviewers upon request. We strongly encourage code deposition in a community repository (e.g. GitHub). See the Nature Research guidelines for submitting code & software for further information.

## Data

Policy information about availability of data

All manuscripts must include a data availability statement. This statement should provide the following information, where applicable:

- Accession codes, unique identifiers, or web links for publicly available datasets
- A list of figures that have associated raw data
- A description of any restrictions on data availability

The data sets generated and analysed during the current study are available from the corresponding author on reasonable request.

# Field-specific reporting

Please select the best fit for your research. If you are not sure, read the appropriate sections before making your selection.

☐ Life sciences ☒ Behavioural & social sciences

For a reference copy of the document with all sections, see nature.com/authors/policies/ReportingSummary-flat.pdf

# Behavioural & social sciences

## Study design

All studies must disclose on these points even when the disclosure is negative.

| | |
|---|---|
| Study description | Semi-structured interviews were conducted with participants enrolled within a mixed-method feasibility study. Qualitative data was generated and analysed. The mixed methods study included patients presenting to their GP with breathlessness and had not yet received a diagnosis for the underlying cause.<br>The original interview guide was expanded to incorporate the COVID-19 pandemic situation; existing topic areas included experiences of breathlessness , related healthcare, and the larger research study. For this report, only data relevant to the impact of the COVID-19 lockdown experience on the diagnostic process were included. |
| Research sample | Twenty participants from a mixed methods study were interviewed. The participants were from any of the 10 participating GP practices in the mixed methods study. The GP practices were in Leicester and Leicestershire, England. Patients within six months of presenting to their GP with breathlessness and willing to participate in an interview were eligible for this study. Twenty participants were interviewed: 12 female, mean (range) age of 65 (45-89) years. Sixteen participants lived in a rural setting and four in the city, five participants lived alone. All participants were retired except for two participants who remained working throughout the UK 'lockdown' period. The mean (range) number of comorbidities for the patients was 4 (0-10). |
| Sampling strategy | Semi-structured interviews were conducted with 20 participants enrolled within a mixed-method feasibility study: Breathlessness – DiagnosE Early in Primary care (Breathe-DEEP) with eligibility criteria of adults over forty years old, breathlessness for longer than two months, presenting for the first time and with no prior diagnoses accounting for their symptoms. The feasibility trial recruitment started in November 2019 and patients within six months of presenting to their GP with breathlessness and willing to participate in an interview were eligible for this study. This was purposive sampling from a group of patients with chronic breathlessness. |
| Data collection | Interviews were conducted via telephone by one of two interviewers, who were trained in qualitative research methods. The interviews were recorded using a Dictaphone and transcribed verbatim. The transcripts were evaluated using thematic analysis supported by NVivo software. |
| Timing | Participants were interviewed between April - May 2020. Figure 1 within the manuscript demonstrates the timeline of the interviews in relation to the UK lockdown for COVID-19. As highlighted in Figure 1 two participants were interviewed shortly after lockdown was eased as their diagnostic process was potentially impacted. No distinct new codes were identified |
| Data exclusions | The interview topic guide included experiences of breathlessness , related healthcare, and the larger research study. For this report, only data relevant to the impact of the COVID-19 lockdown experience on the diagnostic process were included. |
| Non-participation | No participants declined to take part in the interviews. |
| Randomization | Participants were not randomised to experimental groups. The wider study included cluster randomisation by GP practice. |
